# The type 2 diabetes-specific dementia risk score (DSDRS) is associated with frailty, cognitive and functional status amongst Mexican community-dwelling older adults

**DOI:** 10.1186/s12877-020-01776-5

**Published:** 2020-09-22

**Authors:** Omar Yaxmehen Bello-Chavolla, Carlos Alberto Aguilar-Salinas, José Alberto Avila-Funes

**Affiliations:** 1 Dirección de Investigación, Instituto Nacional de Geriatría, Mexico City, Mexico; 2 Unidad de Investigación de Enfermedades Metabólicas, Instituto Nacional de Ciencias Médicas y Nutrición Salvador Zubirán, Vasco de Quiroga 15. CP 14080, Tlalpan, Mexico City, Mexico; 3 Department of Physiology, Facultad de Medicina, Universidad Nacional Autónoma de Mexico, Mexico City, Mexico; 4 Departamento de Endocrinología y Metabolismo, Instituto Nacional de Ciencias Médicas y Nutrición Salvador Zubirán, Mexico City, Mexico; 5 Instituto Tecnologico y de Estudios Superiores de Monterrey Tec Salud, Nuevo León, Mexico; 6 Geriatrics Department, Instituto Nacional de Ciencias Médicas y Nutrición Salvador Zubirán, Mexico City, Mexico; 7 Centre de recherche INSERM, U1219, F-33076 Bordeaux, France

**Keywords:** Diabetes, Dementia, Frailty, Disability, DSDRS

## Abstract

**Background:**

The type 2 diabetes (T2D) specific dementia-risk score (DSDRS) was developed to evaluate dementia risk in older adults with T2D. T2D-related factors have been shown increase the risk of age-related conditions, which might also increase dementia risk. Here, we investigate the associations of DSDRS with frailty, disability, quality of life (QoL) and cognition in community-dwelling older adults with T2D.

**Methods:**

We included 257 community-dwelling older adults with T2D to evaluate the association between DSDRS and Mini-mental state examination (MMSE), Isaac’s set-test (IST), clock drawing test (CDT), quality of life (SF-36), risk of malnutrition (Mini-Nutritional Assessment or MNA), as well as frailty, Katz’ and Lawton-Brody scores. We also assessed the phenotype and correlates of high-estimated dementia risk by assessing individuals with DSDRS >75th age-specific percentiles.

**Results:**

Mean age of participants was 78.0 ± 6.2 years. DSDRS showed a significant correlation with MMSE test, IST, CDT, SF-36, MNA, Lawton-Brody and Katz scores, and an increasing number of frailty components. DSDRS was higher among frail, pre-frail, and subjects with limited ADL and IADL (*p* < 0.001). Participants with DSDRS >75th age-specific percentiles had lower education, MMSE, IST, SF-36, MNA, Katz, Lawton-Brody, and higher frailty scores. High-estimated 10-year dementia risk was associated with ADL and IADL disability, frailty and risk of malnutrition. When assessing individual components of DSDRS, T2D-related microvascular complications were associated to all outcome measures.

**Conclusion:**

The DSDRS is associated with frailty, disability, malnutrition and lower cognitive performance. These findings support that T2D-related factors have significant burden on functional status, QoL, disability and dementia risk.

## Background

Type 2 diabetes mellitus (T2D) is a main cause of morbidity and mortality in the Western World; the highest prevalence of T2D cases occurs in individuals over 60 years in whom it contributes to premature mortality and disability [1]. Consistent epidemiological evidence has shown an increased risk of incident all-cause, vascular and Alzheimer’s disease dementia in individuals with T2D [2, 3]. Screening of dementia risk has gained interest recently, particularly upon identification of modifiable risk factors for dementia to design strategies aimed at delaying or preventing disease onset [4]. Risk stratification in individuals with T2D might be particularly useful, since dementia in individuals with T2D has an earlier onset, thus having significant impact on function and cognition in older adults [5].

Recently, the diabetes-specific dementia risk score (DSDRS) was developed to evaluate dementia risk in American older individuals with T2D. DSDRS predicts of all-cause dementia by evaluating diabetes-specific risk factors including microvascular complications, hyperglycemic and hypoglycemic crises and diabetic foot along with traditional risk factors for dementia such as age, schooling, cardiovascular disease, and depression [6]. Accumulated risk attributable to T2D-related factors has also been independently associated to age-related conditions including frailty, disability and cognitive impairment [7]. In addition, disability, frailty and cognitive impairment are associated to risk of malnutrition, late-life depression and functional impairment, leading to increase dependence and decreasing quality of life besides increasing dementia risk [8, 9]. Therefore, the clinical utility of screening individuals using DSDRS might be approached with the aim of designing specific treatment regimens to improve quality of life (QoL) and functional status in at-risk older adults [10]. Furthermore, since impaired cognition and dementia have significant negative impacts on T2D self-care, closer attention might be given to individuals identified at higher baseline risk [11].

Despite the practicality of the score, the functional and cognitive phenotype identified by the score has not been described beyond clinical features related to T2D. We hypothesized that individuals with higher DSDRS would have functional and cognitive impairment, which are likely to also impact T2D self-care and QoL. Therefore, the main objective of the present study was to determine the associations of the DSDRS with frailty, disability, and cognitive measures aiming to identify the cross-sectional phenotype which relates it to conditions linked to high-dementia risk in subjects with T2D.

## Methods

### Study population

We performed a cross-sectional study of 257 older adults aged ≥70 years who participated in the Coyoacán Cohort, an observational study conducted in the Coyoacán borough located in Southern Mexico City conducted between 2008 and 2009. Complete methodological details for the design and protocols of this study have been published elsewhere, and questionnaire materials were similar for the full cohort [7, 12]. Briefly, recruited participants were non-institutionalized older adults with residence in Coyoacán who were registered at the comprehensive “Food Support, Medical Care and Free Drugs Program” (FMDP) government program. All participants underwent face-to-face interviews to collect self-reported socio-demographic and health data and a comprehensive geriatric assessment which included physical performance tests, cognitive, nutritional, and medical assessment. Baseline questionnaire data were collected between April–May 2008; clinical evaluation and biological sample collection were carried out between June 2008 and July 2009. In the original cohort, a sample of 1294 participants was calculated to ensure a sample size which could estimate a prevalence of ~ 14% of frailty with α = 0.05 and β = 0.20. Among contacted potential participants, acceptance rate was 86.9% and a total of 1124 participants completed the initial interview, which included individuals with and without T2D. For the present study, we included participants with T2D without previous clinical diagnosis of dementia, defined as self-report of previously diagnosed T2D and/or self-report of taking T2D medications (*n* = 236). To account for subjects who were not previously diagnosed with T2D, we included subjects with fasting glucose ≥126 mg/dL (*n* = 21), who had enough information for their dementia-risk stratification using the DSDRS (overall, *n* = 257).

### Definitions for potential correlates with DSDRS

#### Frailty

We used a modified definition of the one proposed by Fried et al. which was previously validated for this population [13, 14]. This modified definition uses data from questionnaires and self-report to define the following dominions, previously described as: a) Unintentional weight loss ≥5 kg in the last 12 months, b) Exhaustion, c) Low physical activity, d) Slowness, and e) Weakness. Participants were categorized as frail if they fulfilled ≥3 criteria, pre-frail if they fulfilled 1–2 criteria, and non-frail if none.

#### Depressive symptoms

The presence of depressive symptoms was defined as a score > 5 in the 15-item version of the Geriatric Depression Scale (GDS).

#### Cognitive performance

Cognitive evaluation comprised an interview-based assessment, which included a questionnaire-based cognitive evaluation comprising Mini-Mental State Examination (MMSE) evaluation, verbal fluency abilities with the Isaacs Set Test (IST) where four semantic categories were successively used (cities, fruits, animals, and colors) and the Clock-drawing test to assess visuo-constructional abilities. Low cognitive performance was based on a modified definition by Blaum et al., defined as scores <25th percentile in both MMSE and the IST semantic verbal fluency test or clock-drawing test, adjusted for sex, age, and schooling based on normative cutoffs previously validated in the Coyoacán Cohort Study to account for inter-ethnic influences on MMSE scores which impact on the sentitivity of the test to detect cognitive impairment [12, 15].

#### Disability

Determined using Lawton-Brody Instrumental Activities of Daily Living (IADL) scale and Katz Index for the Activities of Daily Living (ADL). We defined ADL or IADL disability as having at least one impaired dominion in the Katz scale (ADL disability) or the Lawton-Brody scale (IADL disability) [16–18].

#### Risk of malnutrition

Assessed by the Mini-Nutritional Assessment (MNA) questionnaire, scores < 24 were indicative of at-risk of malnutrition.

#### QoL

Assessed using the self-administered generic instrument SF-36 health questionnaire in the translated and validated version for Mexican population. Items are formulated as statements to evaluate eight specific health scales including physical functioning, physical pain, role limitations due to physical health problems, role limitations due to personal or emotional problems, emotional well-being, social functioning, energy/fatigue and general health perceptions. Scales were classified in two physical (PCS) and mental component scores (MCS).

### Dementia risk calculation

We evaluated dementia risk using the DSDRS [6]. Self-reported variables included in the score considered duration of T2D from diagnosis in years, self-report of diabetic kidney disease (DKD) and diabetic retinopathy, history of insulin use or oral T2D treatment, previous diagnosis of diabetic foot or peripheral vascular disease, acute myocardial infarction, and stroke. Microvascular complications to estimate DSDRS considered the clustering of DKD, and/or diabetic retinopathy; this definition was also used in linear and logistic regression models. Acute metabolic event was defined as a previous episode of hyperglycemia which required hospitalization or hypoglycemia, defined by self-reported episodes of hyperglycemia or fasting glucose levels < 70 mg/dL as recommended by ADA guidelines. High dementia-risk was defined as an estimated 10-year dementia risk >75th age-specific percentile based on incremental 5-year age categories described by the DSDRS. The selection of this age-specific percentile cut-off was performed to provide fair comparisons of DSDRS to detect age-related phenotypes without a significant influence from age as considered in the DSDRS, whilst still allowing a detection of high-risk individuals within each age group.

### Anthropometric and biochemical evaluation

We calculated the body mass index using anthropometric evaluation using the formula of weight in kilograms divided by height in m^2^. Blood samples were acquired after a 10–12 h fast to measure fasting glucose (Yellow Springs Instruments Co.); serum lipid concentrations assessed total cholesterol, triglycerides and HDL-C and were measured using colorimetric assays (Unicel DxC 600 Synchron Clinical System Beckman Coulter).

### Statistical analysis

#### Intergroup differences

We compared groups according to the 75th age-adjusted percentile of DSDRS using Student’s t-test or Mann-Whitney U according to variable distribution. In all descriptive analyses, distribution of categorical variables is reported as frequencies which were compared between groups using chi-squared tests. A *p*-value < 0.05 was established as statistically significant.

#### Correlation between DSDRS, cognitive tests, frailty, and disability components

To investigate the association between dementia risk and the evaluated scores, we tested the correlation of DSDRS with the continuous MMSE, the IST, CDT, MNA, SF-36, Lawton, and Katz scores as well as the number of frailty components using Spearman’s correlation; 95% confidence intervals were estimated using 1000 bootstrap samples. To develop an explanatory model for DSDRS and identify independent predictors for dementia risk using these scores, we used step-wise multiple linear regression analyses adjusted for sex, years of schooling and years since diabetes diagnosis, with model selection carried out using Bayesian Information Criterion (BIC) minimization.

#### Logistic regression analyses

We developed an explanatory model for high-estimated 10-year dementia-risk to investigate the relation of subjects at higher risk with the investigated clinical phenotypes identified by the scores when transformed into categorical variables. For this purpose, we used logistic regression, treating high-dementia risk as the dependent variable and including as predictors frailty, ADL and IADL disability, risk of malnutrition and low cognitive performance; multiple logistic regression was carried out using step-wise models adjusted for years since diabetes diagnosis, years of schooling and sex. Model diagnostics were conducted using R^2^ and the Hosmer-Lemeshow test. Finally, we constructed ROC curves to estimate performance of DSDRS to identify phenotypes of frailty, low cognitive performance, ADL and IADL disability using probability estimates from regression modes; we also calculated sensitivity and specificity for each phenotype.

#### Contribution of DSDRS components to the observed associations

To investigate whether the association of DSDRS with cognition, disability, frailty and impaired QoL were driven by factors other than age, we fitted multiple linear regression models to evaluate which components of the DSDRS were primarily associated with the outcomes. Predictors included individual components of the DSDRS, including age, microvascular complications, depression, diabetic foot, acute metabolic events, cardiovascular and cerebrovascular disease. We included as dependent variables scores correlated to DSDRS, which included the frailty score, Lawton, Katz, MMSE, MNA and SF-36 PCS. Model diagnostics were conducted using R^2^ and BIC; multicollinearity was assessed using tolerance and variance inflation factor (VIF). Predictors were tested on homoscedasticity and linearity assumptions; model diagnostics were conducted evaluating normality of residuals. Model parameters are expressed using β-coefficients and 95%CI. All statistical analyses were performed using the SPSS software (Version 22.0), R (Version 3.6.1) and GraphPad Prism (Version 6.0).

## Results

### Study subjects

**Fig. 1:**
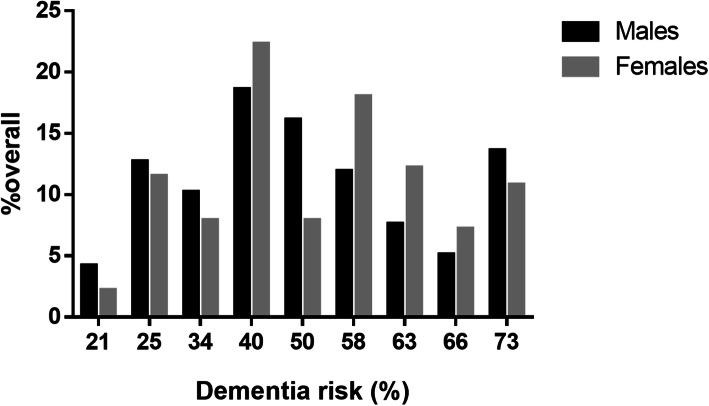
Frequency distribution of estimated 10-year dementia risk amongst Mexican community-dwelling elderly with type 2 diabetes mellitus, stratified by sex. Despite the uneven distribution, we observed no significant differences in sex across dementia risk scores

We included 257 subjects with T2D, with a slight female predominance (54.1%), an average age of 78.0 ± 6.2 years and a median of 10 years since T2D diagnosis. Insulin use was observed in 58 subjects (22.6%), 89 subjects had fasting glucose ≤130 mg/dL (34.6%), 89 subjects were categorized as pre-frail (34.6%) and 32 subjects as frail (12.5%). In relation to microvascular complications, 62 subjects referred having a previous diagnosis of diabetic kidney disease (DKD) and 102 referred diabetic retinopathy (39.7%). Furthermore, 88 subjects (34.2%) referred having any degree of diabetic foot disease; acute metabolic events occurred in 19 subjects (7.4%) of whom 7 were categorized ad hypoglycemic events (2.7%) and 12 as hyperglycemic events requiring hospitalization (4.7%, Table 1). The median of the DSDRS was 8.0 (range 6.0–10.0), which corresponds to an estimated 10-year dementia risk of 50% (40.0–63.0%), which was unevenly distributed by sex, without significant differences in sex distribution across dementia risk categories (*p* = 0.327, Fig. 1). When assessing sex-specific differences in DSDRS components, we identified that female participants had less years of education (6.0 [2.0–11.0], *P* = 0.003) and higher but non-significant rates of microvascular complications (55.4% vs 43.2%, *P* = 0.051) and of diabetic foot (39.6% vs. 28.0%, *p* = 0.051) compared to men but significantly lower rates of acute metabolic events (3.6 vs. 11.9%, *p* = 0.012).

**Table 1:** General characteristics of subjects included in the study, as well as a comparison between individuals with DSDRS above and below the 75th age-specific percentile, defined as high 10-year dementia risk. Results are presented as either mean ± SD or Median (IQR), according to variable distributions. *p < 0.05

Parameter	Overall sample (***N*** = 257) Mean ± SD/Median (IQR)	DSDRS ≤ 75th percentile (***N*** = 198) Mean ± SD or Median (IQR)	DSDRS > 75th percentile (***N*** = 59) Mean ± SD or Median (IQR)
Female sex (%)	139 (54.1)	110 (55.6)	29 (49.2)
Age (years)	78.05 ± 6.16	77.83 ± 6.14	78.79 ± 6.22
Years since T2D diagnosis	10.0 (3.0–20.0)	7.5 (2.0–19.0)	17.0 (10.0–23.0)*
Age at T2D diagnosis	64.93 ± 12.56	66.12 ± 12.30	61.01 ± 12.71*
Schooling (years)	6.0 (1.0–9.0)	6.0 (2.0–9.0)	3.0 (0.0–6.0)*
Glucose (mg/dL)	143.86 ± 60.23	139.13 ± 44.58	158.89 ± 93.22
Triglycerides (mg/dL)	185.36 ± 97.09	181.33 ± 97.55	198.15 ± 95.55
HDL-C (mg/dL)	42.05 ± 12.64	42.08 ± 12.97	41.98 ± 11.64
Total Cholesterol (mg/dL)	194.52 ± 42.01	195.12 ± 42.59	192.61 ± 40.50
BMI (kg/m2)	26.99 ± 4.02	27.01 ± 3.95	26.92 ± 4.28
MMSE score	20.83 ± 5.45	21.81 ± 4.88	17.46 ± 5.99*
Isaac’s set test score	23.78 ± 6.87	24.75 ± 6.41	21.58 ± 7.43*
Clock Drawing Test	2.0 (1.0–5.0)	6.0 (2.0–9.0)	7.5 (2.0–19.0)
Geriatric depression scale	2.0 (1.0–4.0)	2.0 (1.0–3.0)	5.0 (3.5–7.0)*
Katz scale	5.21 ± 1.41	5.44 ± 1.16	4.42 ± 1.83*
Lawton scale	5.30 ± 1.28	5.44 ± 1.11	4.83 ± 1.66*
Mini-Nutritional Assessment	24.92 ± 3.29	25.67 ± 2.59	22.53 ± 4.12*
SF-36 PCS	43.53 ± 9.67	44.35 ± 9.71	39.87 ± 8.68*
SF-36 MCS	52.64 ± 9.59	53.66 ± 8.94	48.07 ± 11.10*
Frailty (%)	32 (15.0)	20 (11.5)	12 (30.8)*
Insulin use (%)	58 (22.6)	46 (23.2)	12 (20.3)
Stroke (%)	20 (7.8)	4 (2.0)	16 (27.1)*
Myocardial infarction (%)	27 (10.5)	14 (7.1)	13 (22.0)*
Acute metabolic events (%)	19 (7.4)	7 (3.5)	12 (20.3)*
Microvascular complications (%)	128 (49.8)	73 (36.9)	55 (93.2)*
Diabetic foot (%)	88 (34.2)	52 (26.3)	36 (61.0)*
DSDRS	8.0 (6.0–10.0)	7.0 (6.0–9.0)	11.0 (9.00–12.0)*

***Abbreviations*:** *T2D* Type 2 diabetes, *DSDRS* Diabetes-specific dementia risk score, *HDL-C* High-density lipoprotein cholesterol, *BMI* Body-mass index, *MMSE* Mini-mental state examination, *ADL* Activities of daily life, *IADL* Instrumented activities of daily life

### DSDRS, cognition, frailty, QoL and functional scores

**Fig. 2:**
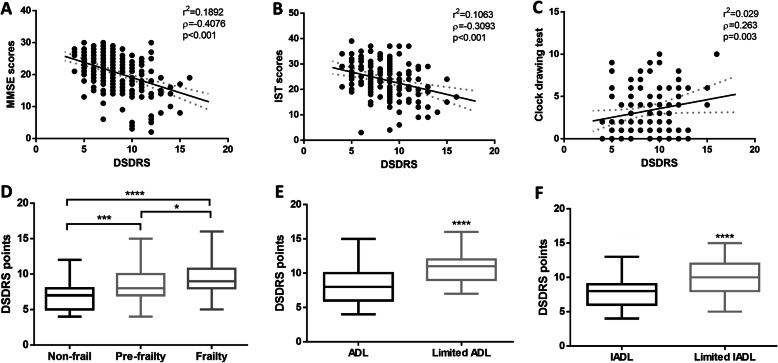
Correlation between increasing DSDRS and MMSE **a**, IST **b** and Clock test scores **c**. We also show comparisons of DSDRS according to frailty categories **d**, and functional status regarding activities of daily life **e** and instrumented activities of daily life **f**, demonstrating the role of DSDRS to discriminate functional and cognitive status. *p < 0.05, **p < 0.01, ***p < 0.001, ****p < 0.0001. Abbreviations: DSDRS, Diabetes-specific dementia risk score; MMSE, Mini-mental state examination; IST, Isaac’s set-test; ADL, Activities of daily life; IADL, Instrumented activities of daily life

We observed negative adjusted and unadjusted linear associations between DSDRS and MMSE, IST, SF-36 PCS, SF-36 MCS and MNA. We also observed a positive and significant linear associations between DSDRS and CDT, Katz, Lawton and the frailty score (Table 2). We did not observe a significant correlation between DSDRS, fasting glucose, triglycerides, HDL-C, total cholesterol or BMI. Using step-wise linear regression analyses we identified that frailty (β = 0.400, 95%CI 0.080–0.719), MMSE (β = − 0.153 95%CI -0.255 - -0.050) and MNA scores (β = − 0.160 95%CI -0.283 - -0.037) explained 26.8% of the variability in DSDRS, adjusted for sex, years of schooling and years since T2D diagnosis (R^2^ = 0.268, *p* < 0.001). Furthermore, we observed significantly higher DSDRS among frail participants compared with non-frail subjects and in subjects with disability (Fig. 2).

**Table 2:** Partial correlation analyses and multiple linear regression of DSDRS with evaluated scores in the sample, adjusted for sex, years of schooling and years since diabetes diagnosis

Parameter	Unadjusted correlation (95%CI)	Adjusted correlation (95%CI)	Multiple Linear R^**2**^
MMSE score	−0.412 (−0.511 - -0.293)	−0.359 (−0.445 - -0.277)	0.329
IST score	−0.319 (− 0.470 - -0.161)	− 0.250 (− 0.421 - -0.111)	0.158
Clock Drawing Test	0.285 (0.114–0.454)	0.257 (0.077–0.434)	0.170
SF-36 PCS	−0.363 (− 0.482 - -0.254)	−0.233 (− 0.361 - -0.117)	0.146
SF-36 MCS	−0.176 (− 0.323 - -0.036)	−0.183 (− 0.319 - -0.082)	0.067
Mini-nutritional assessment	−0.354 (− 0.492 - -0.194)	−0.326 (− 0.457 - -0.168)	0.240
Katz score	−0.340 (− 0.446 - -0.226)	−0.269 (− 0.370 - -0.167)	0.152
Lawton score	−0.217 (− 0.342 - -0.078)	−0.314 (− 0.430 - -0.173)	0.136
Frailty components	0.399 (0.282–0.509)	0.263 (0.077–0.428)	0.191

***Abbreviations*:** *DSDRS* Diabetes-specific dementia risk score, *MMSE* Mini-mental state examination, *IST* Isaac’s Set Test, *MNA* Mini-nutritional assessment, *SF-36 MCS* Mental Component Score of the SF-36 quality of life questionnaire, *SF-36 PCS* Physical Component Score of the SF-36 quality of life questionnaire

### DRDS, decreased cognitive performance, frailty, and disability

Subjects with high 10-year dementia risk had less years of education, lower MNA, SF-36 PCS, SF-36 MCS, MMSE, IST, Katz and Lawton-Brody scores, higher frailty and GDS scores, and years of T2D exposure in comparison to DSDRS <75th age-specific percentile (Table 1). As expected, subjects with high 10-year dementia risk were also more likely to be frail, at risk of malnutrition (50.0% vs. 20.0%, *p* < 0.001), with disability (ADL 29.8% vs. 12.4%, *p* = 0.002; IADL 22.0% vs. 7.1%, *p* = 0.001) and a trend towards low cognitive performance (20.0% vs. 8.8%, *p* = 0.057). When evaluating the role of the DSDRS score to identify frailty and disability we observed highest area under the curve (AUC) for detection of ADL disability (AUC 0.803 95%CI 0.715–0.892; sensitivity 85.7%, specificity 61.3%), IADL disability (AUC 0.733 95%CI 0.629–0.838; sensitivity 62.0%, specificity 75.7%), low cognitive performance (AUC 0.704 95%CI 0.582–0.826; sensitivity 83.3%, specificity 54.3%), and frailty (AUC 0.700 95%CI 0.601–0.799; sensitivity 71.9%, specificity 69.1%). Using logistic regression, high-estimated 10-year dementia risk was associated to ADL and IADL disability, frailty and risk of malnutrition, adjusted for sex, years of schooling and years since T2D diagnosis. In multivariable logistic regression analyses, frailty and risk of malnutrition were associated with high-estimated 10-year dementia risk, adjusted for sex, years of schooling and years since T2D diagnosis (R^2^ = 0.294, *χ*^2^ =8.682, *p* = 0.370, Table 3).

**Table 3:** Simple and multiple logistic regression analyses of the association of evaluated scores and phenotypes with high-estimated dementia risk, defined as DSDRS >75th age-specific percentiles. Analyses were adjusted for sex, years of schooling and years since diabetes diagnosis

	Model diagnostics	Parameter	OR (95%CI)	p-value
Simple	R^2^ = 0.108; *χ*^2^ =9.418, *p* = 0.308	IADL disability	2.92 (1.25–6.85)	0.014
	R^2^ = 0.214; *χ*^2^ =3.113, *p* = 0.927	ADL disability	2.52 (1.15–5.51)	0.021
	R^2^ = 0.063; *χ*^2^ =22.772, *p* = 0.004	Low cognitive performance	2.61 (0.94–7.20))	0.064
	R^2^ = 0.240; *χ*^2^ =4.254, *p* = 0.834	Frailty	3.91 (1.51–10.12)	0.005
	R^2^ = 0.249; *χ*^2^ =7.161, *p* = 0.519	Risk of malnutrition	3.35 (1.39–8.09)	0.007
Multiple	R^2^ = 0.294; *χ*^2^ =8.682, p = 0.370	Frailty	4.31 (1.34–13.94)	0.049
		Risk of malnutrition	2.56 (1.01–6.54)	0.015

***Abbreviations*:** *DSDRS* Diabetes-specific dementia risk score, *MMSE* Mini-mental state examination, *ADL* Activities of daily life, *IADL* impaired activities of daily life

### Specific DSDRS components and outcomes

In the case of the frailty score, age, microvascular complications, diabetic foot, stroke and depression explain the observed associations. Older age, microvascular complications and cardiovascular disease were associated with Lawton scores whilst older age, microvascular complications and were associated with lower Katz scores. For MMSE we observed significant associations with age, schooling, depression and microvascular complications, whilst for MNA scores, we observed associations with diabetic foot disease, cardiovascular disease, stroke and depression. Finally, lower SF-36 PC scores were associated with microvascular disease, cardiovascular disease and depression (Supplementary Material).

## Discussion

Here, we demonstrate that the DSDRS is associated with measures of cognitive performance, frailty, risk of malnutrition, QoL, and ADL/IADL disability among community-dwelling older adults with T2D. Furthermore, we observed higher DSDRS in pre-frail and frail participants and among those with disability. Relying on these associations, the identified cross-sectional phenotype observed using the DSDRS is consistent with what would be expected for patients at higher risk of dementia, regardless of T2D status. Subjects at higher risk of dementia identified by the DSDRS would most likely be frail, have some degree of disability, decreased cognitive performance, risk of malnutrition and lower QoL. These findings strengthen the notion that T2D and T2D-related complications have significant burden on functional status, QoL, disability and, subsequently, on dementia risk.

### Frailty and dementia risk by DSDRS

Older adults with T2D are at an increased risk of frailty; furthermore, interactions between frailty and hypoglycemia during T2D treatment have been reported to increase dementia-risk. This is significant, since patients with increasing number of macro and microvascular complications might be assessed as requiring more intensive glycemic control which, in subjects with impaired functional status and frailty might increase dementia risk [19]. The role of frailty in increasing morbidity and impacting QoL in patients with T2D has previously been shown and has led to recommendations against intensive glycemic control in this population [20, 21]; in addition, frailty is related to vascular damage and might contribute to increased risk of vascular dementia in T2D [22]. DSDRS might prove useful to identify patients with impaired functional status, multiple comorbidities, and frailty, who might benefit from less intensive T2D treatment and might require treatment adjustments [21]. In our study, we did not assess hypoglycemic episodes or hypoglycemia risk, but the interaction between frailty and hypoglycemia in relation to DSDRS and its impact on dementia risk should be evaluated in future studies.

### Microvascular complications and dementia risk by DSDRS

Microvascular complications, particularly diabetic retinopathy, are evaluated by DSDRS to discriminate subjects with increased dementia risk who might also have disability, impaired QoL and functional status [23]. Diabetic retinopathy and neuropathy cause severe sensory impairments in older patients with T2D; furthermore, T2D has been associated to increased risk of bilateral sensorineural hearing loss in addition to established microvascular damage, which might contribute to further sensory loss [24, 25]. Sensory impairments, particularly in eyesight, hearing, and neuropathy have been associated with increased dementia risk and favor the development of disability and increased mortality; in addition, end-organ microvascular damage in T2D increases risk of falls and impairment of functional status [26, 27]. The role of microvascular damage in the pathophysiology of dementia in diabetes has also been studied, but the evidence of this association is inconsistent and neurological changes have been observed in individuals with T2D without end-organ microvascular damage [28]. Thus, DSDRS might identify individuals at high risk of disability in IADL and ADL due in part to sensory impairment by recognizing a population with increased dementia risk in whom rehabilitation would be beneficial [29]. The role of depression, macro and microvascular factors and obesity in promoting disability in individuals with T2D has also been reported, and its evaluation by the score contributes to the identification of individuals with ADL and IADL disability [30].

### Cognition, microvascular complications and DSDRS

Older adults with T2D present lower performance on cognitive evaluations, particularly when affected by micro and macrovascular complications [31]. Cognitive evaluations of individuals with T2D have demonstrated impaired domains in information processing speed, visuospatial functions, attention, executive functioning abstract reasoning [32]; furthermore, individuals with T2D present a higher rate of cognitive decline, directly dependent with glycemic control. Individuals with T2D and cognitive impairment also experience a higher rate of conversion to dementia, with earlier disease onset and increased disease progression in relation to T2D duration and microvascular complications, as has been shown in previous studies [33, 34]. The lower cognitive performance observed in individuals with increased DSDRS in our study might be attributable to the consideration of microvascular damage, age and glycemic control, which underlie associations with impaired cognition in T2D in most prospective studies. Furthermore, the impact of frailty and disability on cognition, both of which increase dementia risk, must also be considered [35, 36]. Since individuals with T2D are a population with high susceptibility to impaired cognition, our demonstration of lower cognitive performance when screening subjects using the DSDRS provides evidence for its utility in a cross-sectional setting. Future longitudinal studies should shed light on the role of DSDRS for prediction of cognitive impairment conversion to dementia in individuals with T2D.

### Strengths and limitations

Our study had some strengths and limitations. First, we performed a wide range of evaluations, which allowed a thorough assessment of cognition, QoL, disability, and frailty in a sample of community-dwelling individuals with T2D in which most of these predictors had previously been validated. We also demonstrated that the DSDRS identifies subjects who could be considered for short-term interventions to improve function and ameliorate the negative effects of disability, frailty and T2D related complications. Furthermore, this is the first study in which DSDRS is used besides its original evaluation, demonstrating its utility in different populations and settings. Validation of DSDRS for functional and frailty status as demonstrated in this study allows to characterize the phenotype observed for subjects with high-estimated dementia risk score and extend the applications of DSDRS; nevertheless, prospective validation studies are required to both externally validate the score in different populations and replicate the observed associations for DSDRS in this study.

Amongst the limitations to be acknowledged is its cross-sectional setting, which limits the ability to establish causal relationships and the self-report of comorbidities and T2D complications, which might underestimate the true impact of the associations. The modified frailty definition which uses self-reported measures instead of physical evaluations has previously been applied in other studies involving the Coyoacán Cohort study; furthermore, epidemiological studies in other settings with self-reported data have been conducted and yielded reproducible results [17, 37]. Recent studies have compared the performance of frailty definitions which substitute physical based for self-reported based measures, identifying adequate diagnostic performance for self-reported definitions and adequate concordance with physical based definitions [38, 39]. Given this evidence, we propose that this operational definition should adequately capture the variability of the frailty phenotype in our subjects. Since the evaluated sample was representative of the community and previous reports have shown an increased rate of undiagnosed T2D in our population [1], some cases of T2D with fasting glucose < 126 mg/dL could have been excluded, thus limiting information of such cases. In addition, since no specific dementia information was available for diagnosis in the Coyoacán Cohort study there exists a possibility for undiagnosed cases of dementia not identified by cognitive assessment, thus modifying the strength of these associations. Our results however are in accordance with recent findings which show that DSDRS might be useful to detect cognitive impairment in indivudals with T2D [40]. Moreover, cognitive assessment did not include more extensive measures of executive function, which are highly sensitive to T2D-related cognitive changes and remain to be explored in future studies.

## Conclusions

The DSDRS is associated with frailty, disability, risk of malnutrition, lower cognitive performance and impaired quality of life. Evaluation of this score in primary care facilities might prove useful for identification of subjects with T2D who might benefit from multidisciplinary interventions focusing on rehabilitation to improve upon IADL and ADL disability, frequent cognitive screening, nutritional counseling and evaluation of interventions to reduce burden related to frailty. The role of said interventions to delay onset of cognitive decline and dementia in high risk patients identified using the DSDRS should be evaluated in future studies.

## Supplementary information

**Additional file 1.** Supplementary Material. Regression models for individual components of DSDRS and its prediction of age-related phenotypes. This section includes an additional table which summarizes regression models for prediction of age-related phenotypes.

## Data Availability

The datasets used and/or analysed during the current study are available from the corresponding author on reasonable request.

## Abbreviations

T2D: Type 2 diabetes
DSDRS: Diabetes-specific dementia risk score
HDL-C: High-density lipoprotein cholesterol
BMI: Body-mass index
MMSE: Mini-mental state examination
ADL: Activities of daily life
IADL: Instrumented activities of daily life
MMSE: Mini-mental state examination
IST: Isaac’s Set Test
MNA: Mini-nutritional assessment
SF-36 MCS: Mental Component Score of the SF-36 quality of life questionnaire
SF-36 PCS: Physical Component Score of the SF-36 quality of life questionnaire
