# Supplementary material for: The type 2 diabetes-specific dementia risk score (DSDRS) is associated with frailty, cognitive and functional status amongst Mexican community-dwelling older adults
Source: BMC Geriatr. 2020 Sep 22;20:363. doi: 10.1186/s12877-020-01776-5 (PMC7510254; doi:10.1186/s12877-020-01776-5)
Supplement: Supplementary file 1 — Additional file 1. Supplementary Material. Regression models for individual components of DSDRS and its prediction of age-related phenotypes. This section includes an additional table which summarizes regression models for prediction of age-related phenotypes. [file 12877_2020_1776_MOESM1_ESM.docx]

**Supplementary Table 1.** Linear regression analyses of the association of individual DSDRS components with each evaluated score to assess individual contributions to their associations with DSDRS in community-dwelling older adults.

| Model | Parameter | β-coefficient | t | 95%CI | p-value |
| --- | --- | --- | --- | --- | --- |
| Frailty score  r^2^=0.203, p<0.001 | Age | 0.110 | 2.167 | 0.10-0.210 | 0.031 |
|  | Microvascular complications | 0.385 | 2.220 | 0.043-0.727 | 0.027 |
|  | Diabetic foot | 0.368 | 2.048 | 0.014-0.722 | 0.042 |
|  | Depressive symptoms | 0.483 | 4.277 | 0.260-0.705) | <0.001 |
|  | Stroke | 0.305 | 1.985 | 0.002-0.608 | 0.048 |
| Lawton-Brody score  r^2^=0.049, p=0.001 | Age | -0.118 | -2.523 | -0.211 - -0.026 | 0.012 |
|  | Microvascular complications | -0.314 | -2.003 | -0.623 - -0.005 | 0.046 |
|  | Cardiovascular disease | -0.496 | -1.935 | -1.001 – 0.009 | 0.054 |
| Katz scores  r^2^=0.165, p<0.001 | Age | -0.161 | -3.232 | -0.259 - -0.063 | 0.001 |
|  | Microvascular complications | -0.612 | -3.665 | -0.941 - -0.283 | <0.001 |
|  | Depressive symptoms | -0.407 | -3.886 | -0.614 - -0.201 | <0.001 |
| MNA scores  r^2^=0.294, p<0.001 | Diabetic foot | -1.284 | -2.737 | -2.211 - -0.357 | 0.007 |
|  | Cardiovascular disease | -3.074 | -3.843 | -4.655 - -1.493 | <0.001 |
|  | Stroke | -1.480 | -3.566 | -2.299 - -0.660 | <0.001 |
|  | Depressive symptoms | -1.221 | -4.340 | -1.777 - -0.665 | <0.001 |
| MMSE scores  r^2^=0.283, p<0.001 | Age | -0.760 | -4.341 | -1.105 - -0.415 | <0.001 |
|  | Microvascular disease | -1.463 | -2.259 | -2.739 - -0.188 | 0.025 |
|  | Schooling | -4.069 | -4.781 | -5.746 - -2.393 | <0.001 |
|  | Depression | -2.200 | -5.848 | -2.914 - -1.459 | <0.001 |
| SF-36 PCS  r^2^=0.142, p<0.001 | Microvascular disease | -5.420 | -4.268 | -7.923 - -2.916 | <0.001 |
|  | Cardiovascular disease | -4.757 | -2.354 | -8.742 - -0.772 | 0.020 |
|  | Depression | -2.371 | -2.642 | -4.140 - -0.602 | 0.009 |

**Abbreviations:** DSDRS, Diabetes-specific dementia risk score; MMSE, Mini-mental state examination; MNA: Mini-nutritional assessment; SF-36 PCS: Physical Component Score of the SF-36 quality of life questionnaire.
